# Secretagogin expression in the mouse olfactory bulb under sensory impairments

**DOI:** 10.1038/s41598-020-78499-5

**Published:** 2020-12-09

**Authors:** L. Pérez-Revuelta, P. G. Téllez de Meneses, M. López, J. G. Briñón, E. Weruaga, D. Díaz, J. R. Alonso

**Affiliations:** 1 Laboratory of Neuronal Plasticity and Neurorepair, Institute for Neuroscience of Castile and Leon (INCyL), University of Salamanca, C/ Pintor Fernando Gallego, 1, 37007 Salamanca, Spain; 2 Institute of Biomedical Research of Salamanca, IBSAL, 37007 Salamanca, Spain

**Keywords:** Olfactory bulb, Adult neurogenesis

## Abstract

The interneurons of the olfactory bulb (OB) are characterized by the expression of different calcium-binding proteins, whose specific functions are not fully understood. This is the case of one of the most recently discovered, the secretagogin (SCGN), which is expressed in interneurons of the glomerular and the granule cell layers, but whose function in the olfactory pathway is still unknown. To address this question, we examined the distribution, generation and activity of SCGN-positive interneurons in the OB of two complementary models of olfactory impairments: Purkinje Cell Degeneration (PCD) and olfactory-deprived mice. Our results showed a significant increase in the density of SCGN-positive cells in the inframitral layers of olfactory-deprived mice as compared to control animals. Moreover, BrdU analyses revealed that these additional SCGN-positive cells are not newly formed. Finally, the neuronal activity, estimated by c-Fos expression, increased in preexisting SCGN-positive interneurons of both deprived and PCD mice -being higher in the later- in comparison with control animals. Altogether, our results suggest that the OB possesses different compensatory mechanisms depending on the type of alteration. Particularly, the SCGN expression is dependent of olfactory stimuli and its function may be related to a compensation against a reduction in sensory inputs.

## Introduction

The main olfactory bulb (OB) is the part of the brain that receives the olfactory signals from the sensory neurons of the olfactory epithelium for processing first-odor information, which is then further completed in the higher olfactory centers. The OB has a well-laminated structure comprised of the superficially-located glomerular layer (GL), the external plexiform layer (EPL), the mitral cell layer (MCL), the internal plexiform layer (IPL), the granule cell layer (GCL) and the most internal tissue comprised of periependymal white matter. There are dual signaling pathways for the initial integrated signals, those that lack inhibition or inhibition-modulated late-refined signals from interneurons, mediated by tufted cells and mitral cells respectively^1^. Also, there are different types and subtypes of interneurons, such as juxtaglomerular cells, granule cells and short axon cells^2,3^, all of which are highly likely to be essential for fine odor discrimination and learning^4,5^; however, their subtype-specific functions are not fully understood. Subtypes of interneurons are usually identified by distinct subsets of several neurochemical markers including calcium-binding proteins.

Secretagogin (SCGN) is a calcium-binding protein of the “EF-hand” family and is one of the most recently discovered molecules of this group^6^. SCGN is widely expressed throughout the OB layers, but it is specifically clustered in the GL, MCL and GCL^7,8^. Concerning the SCGN-positive cell types, they are heterogeneous and comprise numerous juxtaglomerular neurons, granule cells, small to medium-sized neurons in the EPL and a few small cells in the ependymal/subependymal layer^7^.

Although it is known that SCGN can be involved in neuronal differentiation and replacement processes^9^, its function is not fully understood, especially in the olfactory pathway. A classic approach to study the function of this and other proteins and their relationship with the olfactory system is the use of an altered model.

Therefore, in this study we analyzed the expression of SCGN in two complementary animal models with olfactory alterations: (1) olfactory-deprived mice, a sensory model that prevents the arrival of orthonasal olfactory stimuli, although the structural integrity of the central olfactory pathway is physically intact^10,11^; and (2) Purkinje Cell Degeneration (PCD) mice, which suffer degeneration in the main projection neurons of the OB (i.e. mitral cells)^12^ but preserve normal olfactory inputs^13,14^.

Olfactory deprivation is based on preventing the interaction of odorants with olfactory receptor neurons by surgically occluding at least one nostril. After occlusion, the olfactory system is free of afferent orthonasal peripheral stimulation. There is evidence that the lack of sensory stimulation causes different alterations in the olfactory system such as a generalized decrease in neuronal activity or a reduction in size of the ipsilateral OB to the occluded nostril^15^. In terms of cell number, granule cells are the most affected populations^16^ while the mitral and tufted cells are resistant to those changes induced by sensory-deprivation^17^. Moreover, after deprivation, the ipsilateral OB suffers an increase in cell death that is highly significant in both GCL and GL^18^.

In parallel, after deprivation, an accumulation of newly formed cells has been described in the rostral migratory stream, specifically located at the entry of the OB^19^. In addition, there is a decrease in metabolic activity which causes alterations in both genetic expression and protein synthesis. Remarkably, the most pronounced neurochemical change is the reduced number of tyrosine hydroxylase (TH) positive juxtaglomerular cells, the first enzyme in the dopamine biosynthetic pathway^20^; as a result, this decrease is used as a primary marker for deprivation in the OB^15,20,21^. Finally, deprivation causes an alteration in several calcium-binding proteins, whose expression is significantly reduced^22^.

Besides, the OB of PCD mutant mice suffers the degeneration of mitral cells. It starts approximately at postnatal day 60 (P60), becomes evident at P70 and ends around P110, where only a 20% of the original population of these neurons remains alive^23^. The death of mitral cells significantly affects the structural organization of the olfactory system, causing a reduction in the total volume of the OB and affecting all of its layers, but to a much lesser extent the GCL, where the most abundant modulating elements the granule cells are located^13^. In contrast to deprived mice, calcium-binding proteins have less variability in PCD animals^24^.

However, to our knowledge there are no studies that analyze SCGN expression in both deprived and PCD mice. The study of possible variations in both models would help to understand the implication of SCGN in olfactory processing.

## Results

### The number of tyrosine hydroxylase-positive cells were significantly reduced in the GL of deprived mice

**Figure 1:**
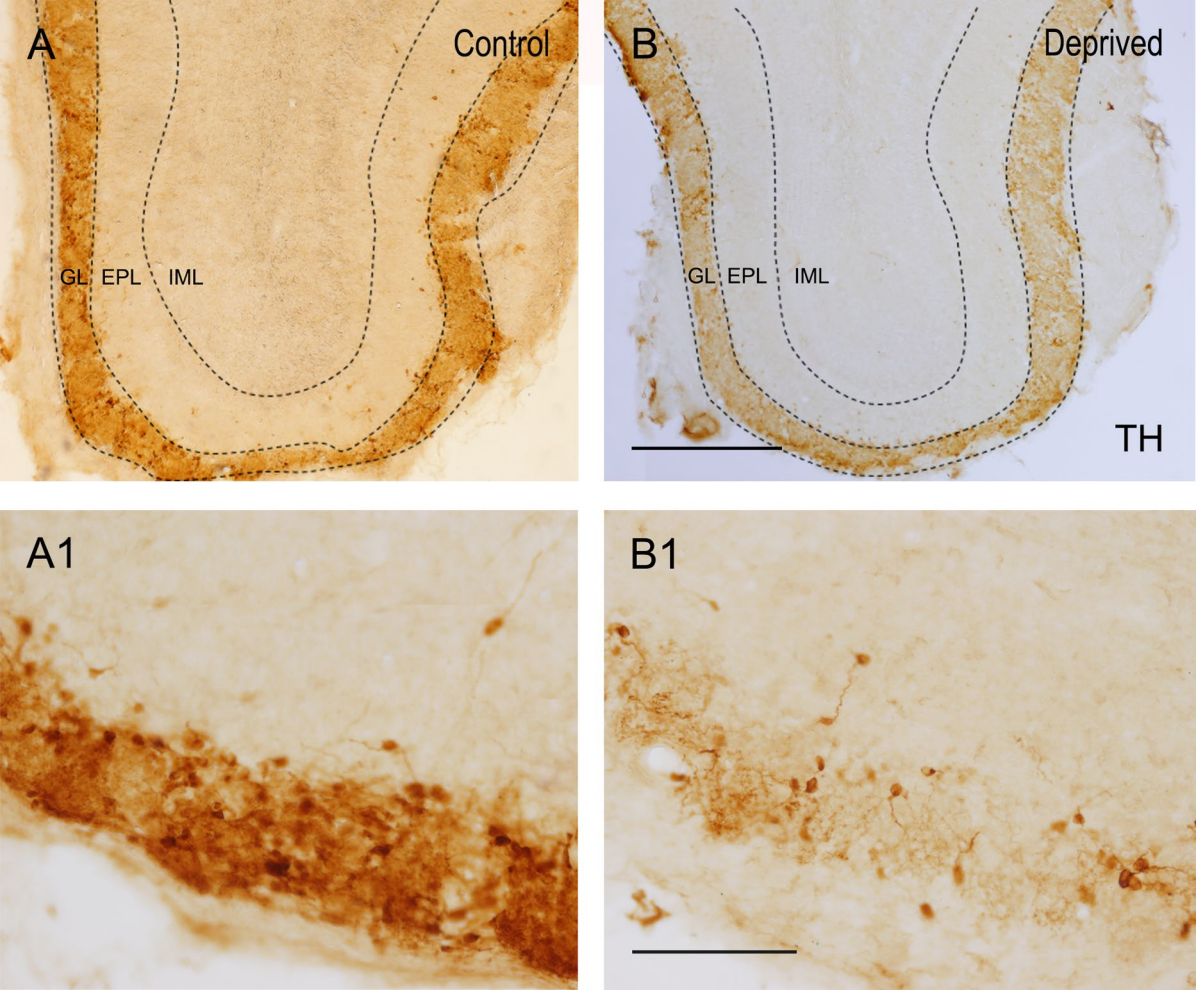
TH expression in the OB of control (**A**,**A1**) and deprived (**B**,**B1**) mice. The images show the juxtaglomerular cells positive for TH. As can be observed, the expression of TH is lower in deprived mice. *GL* glomerular layer, *EPL* external plexiform layer, *IML* inframitral layers. Scale bar (**A**,**B**) 500 µm and (**C**,**D**) 200 µm.

To verify the quality of deprivation, we analyzed TH expression in juxtaglomerular cells. Forty days after the unilateral occlusion, we observed a statistical reduction of 37.63% in TH-positive cells in the deprived mice (8 ± 2 cells per glomerulus, n = 6, rounding the quantities to whole numbers) compared to the control (13 ± 2 TH-positive cells per glomerulus, n = 6, *p* = 0.03, Fig. 1). Therefore, these data confirmed that the deprivation was optimal, allowing us to further analyze the cell subtypes of each of these mice.

### Increase in the density of SCGN-positive cells in the inframitral layers of deprived animals but not in PCD mice

**Figure 2:**
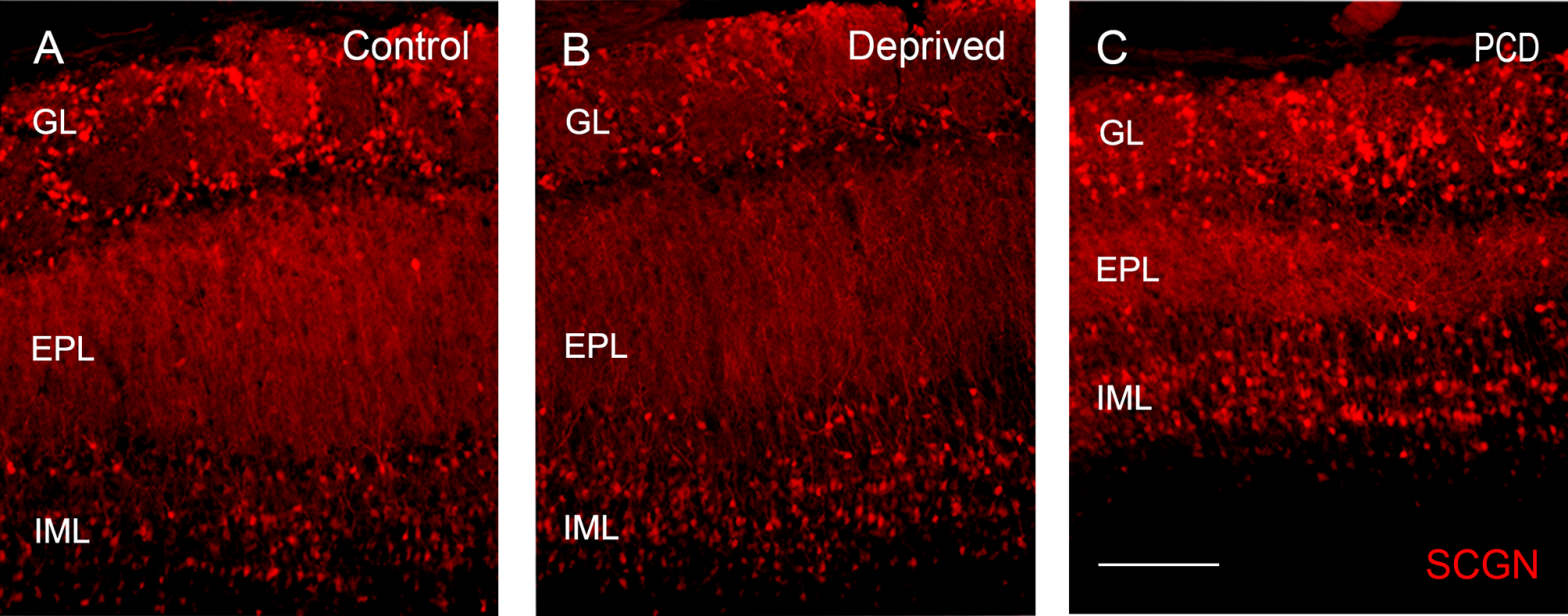
SCGN expression in control (**A**), deprived (**B**) and PCD (**C**) mice. SCGN-positive cells are mainly found in the glomerular (GL) and inframitral layers (IML); in addition, some isolated cells appear in the external plexiform layer (EPL). Note the reduction in the volume of the EPL in PCD animals. Scale bar equals 100 µm.

In the control mice, SCGN-positive cells were observed mainly in the GL and inframitral layers, both at caudal and rostral levels (Fig. 2A). The densities of SCGN-positive cells mostly ranged from 1000 to 2000 cells/mm^2^. In contrast, in the EPL, the cell density decreased by almost one order of magnitude.

**Figure 3:**
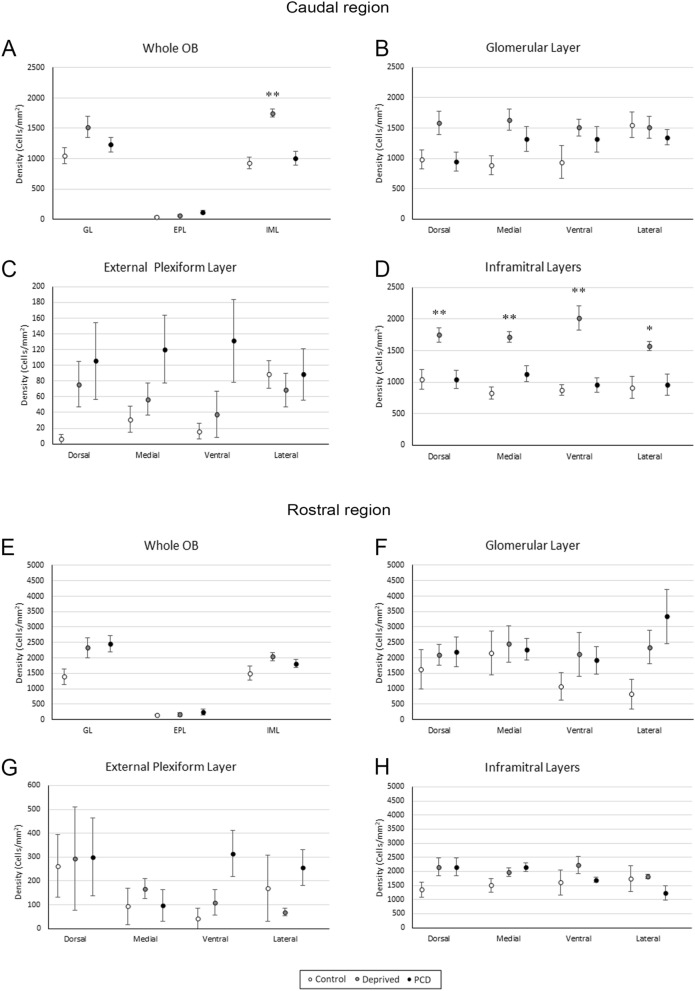
Quantification of SCGN expression in the caudal (**A**–**D**) and rostral levels (**E**–**H**) of the OB. The graphs show the variation in cell density within the glomerular layer (GL), the external plexiform layer (EPL) and inframitral layers (IML) in control (white), deprived (gray) and PCD (black) mice. As can be observed, there are significant variations in the inframitral layers of deprived animals compared to the control mice in the caudal, whereas in the rostral level there are no significant differences. *p < 0.05, **p < 0.01.

In deprived mice, we observed a qualitative increase in the inframitral layers compared to the controls due to the effect of the occlusion. However, GL and EPL showed nonapparent changes in SCGN expression in comparison with their control counterparts (Fig. 2B). After counting, our results showed that SCGN-positive cells markedly increased to the density of 1749 ± 66 cells/mm^2^ (whole OB, n = 6, rounding the quantities to whole numbers) in the caudal sector of the inframitral layers of the OB compared to those of the control mice (930 ± 95 cells/mm^2^, whole OB, n = 6; Fig. 3A, Suppl. Table S1). This increase was statistically significant in the whole OB (Kruskal–Wallis test, *p* = 0.005) and, separately, in the four analyzed sectors: dorsal (1750 ± 113 cells/mm^2^ vs. 1044 ± 156 cells/mm^2^, *p* = 0.009), medial (1714 ± 88 cells/mm^2^ vs. 828 ± 97, *p* = 0.007), ventral (2017 ± 192 cells/mm^2^ vs. 872 ± 87 cells/mm^2^, *p* = 0.008) and lateral (1571 ± 75 cells/mm^2^ vs. 914 ± 172 cells/mm^2^, *p* = 0.025; Fig. 3D, Suppl. Table S1). In sum, deprived mice presented an increase in the density of SCGN-positive cells in the inframitral layers (around 188%) in comparison with the control mice. However, in the EPL we did not find differences in the OB as a whole (59 ± 9 cells/mm^2^ vs. 35 ± 8 cells/mm^2^, *p* = 0.118; Fig. 3A, Suppl. Table S1) or in the four separate sectors: dorsal (76 ± 29 cells/mm^2^ vs. 6 ± 6 cells/mm^2^, *p* = 0.239), medial (57 ± 20 cells^2^ vs. 31 ± 16 cells/mm^2^, *p* = 0.221), ventral (38 ± 29 cells/mm^2^ vs. 16 ± 10 cells/mm^2^, *p* = 0.102) and lateral (69 ± 21 cells/mm^2^ vs. 88 ± 17 cells/mm^2^, *p* = 0.745; Fig. 3C, Suppl. Table S1). Similarly, in the GL, we did not find differences in the OB as a whole (1519 ± 175 cells/mm^2^ vs. 1047 ± 128 cells/mm^2^, *p* = 0.165, Fig. 3A, Suppl. Table S1) or in the four analyzed sectors: dorsal (1581 ± 195 cells/mm^2^ vs. 982 ± 154 cells/mm^2^, *p* = 0.066), medial (1635 ± 173 cells/mm^2^ vs. 886 ± 160 cells/mm^2^, *p* = 0.183), ventral (1507 ± 138 cells/mm^2^ vs. 935 ± 271 cells/mm^2^, *p* = 0.138) and lateral (1511 ± 185 cells/mm^2^ vs. 1553 ± 214 cells/mm^2^, *p* = 0.740; Fig. 3B, Suppl. Table S1). For further information, see Supplementary Table S1.

In PCD mice, the quantity of SCGN-positive cells did not appear qualitatively altered in the caudal section (Fig. 2C). Besides, we observed a size change in the EPL due to the degeneration itself, which modified the anatomy of the OB and the EPL appeared narrower^25^. Moreover, no statistically significant differences were found in inframitral layers either regarding the complete OB (1005 ± 115 cells/mm^2^ vs. 930 ± 95 cells/mm^2^, *p* = 0.831; Fig. 3A, Suppl. Table S1) or in the four sectors considered: dorsal (1047 ± 146 cells/mm^2^ vs. 1044 ± 156 cells/mm^2^, *p* = 0.624), medial (1130 ± 128 cells/mm^2^ vs. 828 ± 97 cells/mm^2^, *p* = 0.136), ventral (955 ± 116 cells/mm^2^ vs. 872 ± 87 cells/mm^2^, *p* = 0.394) and lateral (955 ± 166 cells/mm^2^ vs. 914 ± 172 cells/mm^2^, *p* = 0.831; Fig. 3D, Suppl. Table S1). Likewise, in the EPL, we did not find differences in the OB as a whole (112 ± 31 cells/mm^2^ vs. 35 ± 8 cells/mm^2^, *p* = 0.068) or in the separate sectors: dorsal (106 ± 49 cells/mm^2^ vs. 6 ± 6 cells/mm^2^, *p* = 0.180), medial (121 ± 43 cells/mm^2^ vs. 31 ± 16 cells/mm^2^, *p* = 0.131), ventral (131 ± 53 cells/mm^2^ vs. 16 ± 10 cells/mm^2^, *p* = 0.062) and lateral (89 ± 33 cells/mm^2^ vs. 88 ± 17 cells/mm^2^, *p* = 0.522; Fig. 3C, Suppl. Table S1). Equally, in the GL, we did not observe any statistically significant differences in the whole OB (1231 ± 117 cells/mm^2^ vs. 1047 ± 128 cells/mm^2^, *p* = 0.201), or in the four sectors analyzed: dorsal (946 ± 158 cells/mm^2^ vs. 982 ± 154 cells/mm^2^, *p* = 1), medial (1319 ± 204 cells/mm^2^ vs. 886 ± 160 cells/mm^2^, *p* = 0.136), ventral (1316 ± 211 cells/mm^2^ vs. 935 ± 271 cells/mm^2^, *p* = 0.149) and lateral sectors (1349 ± 130 cells/mm^2^ vs. 1553 ± 214 cells/mm^2^, *p* = 0.522; Fig. 3B, Suppl. Table S1). For further information, see Supplementary Table S1.

Additionally, the rostral level did not present any qualitative differences in SCGN- positive cells either in deprived or PCD mice. The analyses performed in the rostral level were similar to those used to analyze the caudal portion. In this case, the Kruskal–Wallis test showed no statistically significant differences between any experimental group within any of the sectors analyzed (n = 5; *p* > 0.05; Fig. 3E–H; Suppl. Table S1). Therefore, no further analysis was performed.

### Olfactory deprivation did not change the formation of new SCGN-positive cells

Since olfactory deprivation entails a higher density of SCGN-positive cells, we examined whether the additional SCGN-positive cells were generated de novo (i.e. after cell proliferation), with subsequent migration to the caudal region of the IML and GL and then differentiation, or whether they were preexisting cells that started to express the SCGN protein. To address this question, we decided to analyze the differentiation of newly proliferated cells using bromodeoxyuridine (BrdU).

Previous studies carried out in our laboratory showed no alterations in the cell proliferation rate or in the tangential migration through the rostral migratory stream in PCD animals, as compared to the control mice^25^. Furthermore, the expression of other calcium-binding proteins, such as calretinin, parvalbumin and calbindin, did not show changes in the GCL of PCD mutant mice^25^. Similarly, no changes in SCGN-positive cells were detected in PCD animals (see above). Based on these findings, the analysis of BrdU was performed only on deprived and control mice, in order to reduce the number of animals used (2010/63/UE, Recommendation 2007/526/CE; Law 32/2007, RD 53/2013). Therefore, we injected BrdU intraperitoneally in two new sets of animals (n = 5 each), either 15 or 30 days before sacrifice.

**Figure 4:**
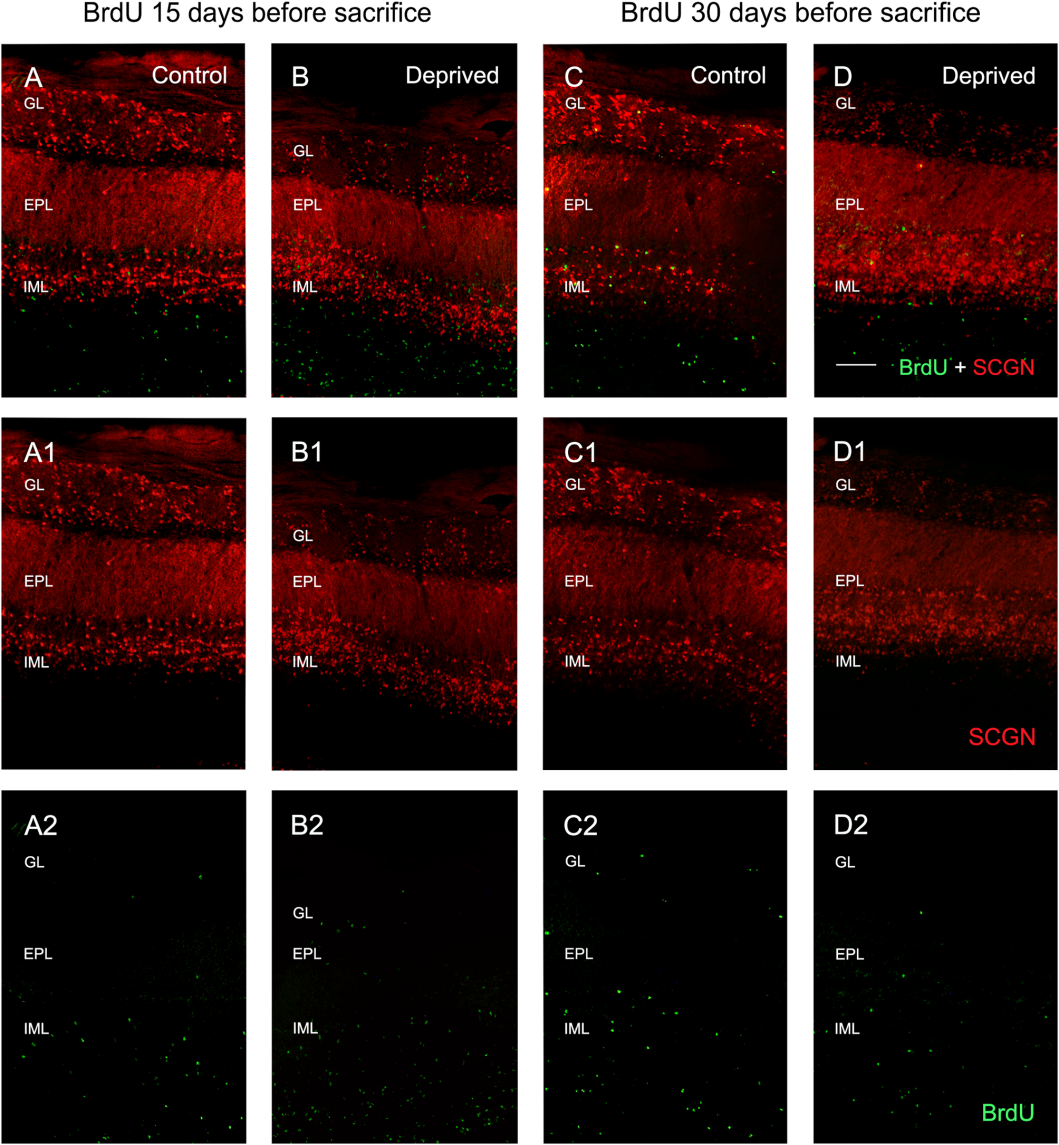
BrdU (green) and SCGN (red) expression in the OB of control (**A**,**C**) and deprived (**B**,**D**) mice. The separate channels are also shown for SCGN (**A1**–**D1**) and BrdU (**A2**–**D2**). Colocalization is scarce when BrdU is injected 15 days before the sacrifice of animals (**A**,**B**), and BrdU-positive cells are mainly found in the innermost OB layers. The analyses of BrdU injection 30 days before sacrifice show an increase in BrdU-positive cells, but BrdU-SCGN colocalization is also extremely low in both control and deprived animals (**C**,**D**). *GL* glomerular layer, *EPL* external plexiform layer, *IML* inframitral layers. Scale bar 100 µm.

In order to analyze the arrival of new cells to the OB, we injected BrdU 15 days before sacrifice, that is to say, 25 days after olfactory deprivation. The cells labeled with this molecule were found, in both deprived and control mice, mainly in the internal layers of the OB, especially in the periependymal white matter and the inframitral layers, while only isolated cells were observed in the rest of the layers (Fig. 4A,B). In addition, very scarce colocalization of SCGN and BrdU was detected in both deprived and control mice, which suggests that BrdU administration 15 days before the sacrifice of animals was not time enough for the neuroblasts to differentiate, express SCGN and fulfill their function in the different OB strata (see discussion).

Therefore, to confirm the stability of BrdU-labeled cells and their differentiation, we injected BrdU 30 days before the animals were sacrificed (10 days after unilateral occlusion). We observed an increase in the number of BrdU-positive cells, especially in the outermost OB layers of both deprived and control mice. However, the BrdU and SCGN colocalization was still quite low (Fig. 4C,D).

**Figure 5:**
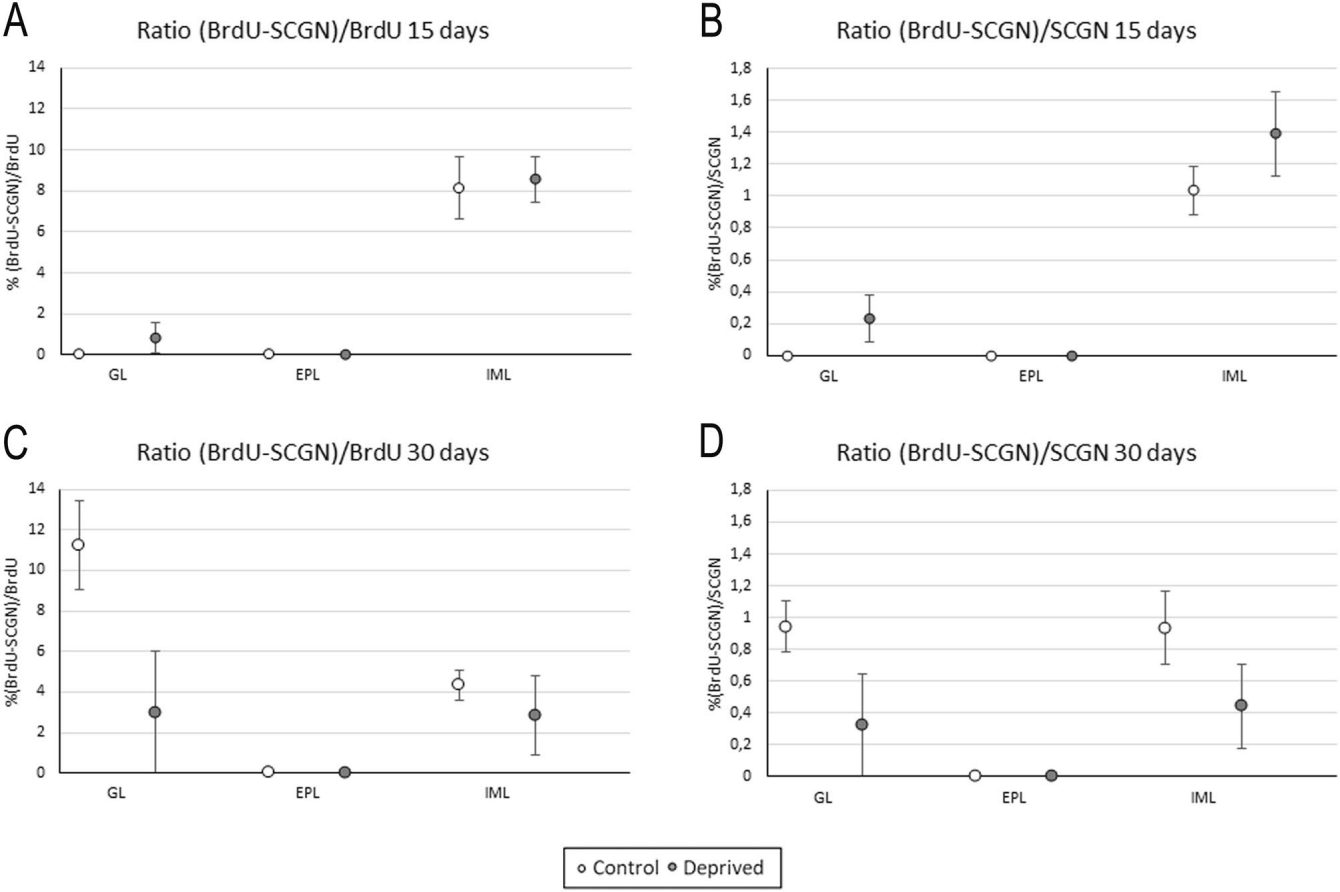
Quantification of BrdU-positive cells 15 (**A**,**B**) and 30 (**C**,**D**) days after BrdU administration in control (white) and deprived (gray) mice. (**A**) and (**C**) represent the ratio of doubly labeled cells in relation to the total BrdU-positive elements, that is, the cells that differentiate into SCGN-expressing neurons; (**B**) and (**D**) represent the ratio of cells doubly labeled in relation to the total SCGN-positive cells, which indicates how many cells of the total population of SCGN are newly formed. Analyses showed no statistically significant differences.

Once the corresponding cell counts were performed, two cell ratios were calculated: (1) the proportion of doubly labeled cells for BrdU and SCGN, in relation to the total number of BrdU-positive elements (BrdU-SCGN/BrdU); and (2) the proportion of doubly labeled cells in relation to the total number of SCGN-positive cells (BrdU-SCGN/SCGN). We did not find statistically significant differences between the deprived and control mice with regard to the BrdU-SCGN/BrdU ratio at 15 days after BrdU injection in either GL (0.83 ± 0.74% vs. 0%, *p* = 0.264), EPL (0% vs. 0%, *p* = 1) or inframitral layers (8.56 ± 1.10% vs. 8.14 ± 1.50%, *p* = 0.754). Similarly, no differences were detected at 30 days after BrdU injection in either the GL (3 ± 3% vs. 11.23 ± 2.17%, *p* = 0.076), EPL (0% vs. 0%, *p* = 1) or inframitral layers (2.84 ± 1.96% vs. 4.32 ± 0.76%, *p* = 0.384; Fig. 5A,C). Concerning the ratios of BrdU-SCGN/SCGN, no statistically significant differences were found between deprived and control mice at 15 days after BrdU injection in GL (0.24 ± 0.14% vs. 0%, *p* = 0.136), EPL (0% vs. 0%, *p* = 1) and inframitral layers (1.39 ± 0.26% vs. 1.03 ± 0.15%, *p* = 0.251) or at 30 days after BrdU injection in GL (0.32 ± 0.32% vs. 0.94 ± 0.16%, *p* = 0.139), EPL (0% vs 0%, *p* = 1) and inframitral layers (0.44 ± 0.26% vs. 0.94 ± 0.22%, *p* = 0.245; Fig. 5B,D).

That is to say, deprivation did not affect the number of newly formed cells that differentiate into SCGN-positive neurons (BrdU-SCGN/BrdU) or the newly formed SCGN-positive cells that are included in the total SCGN-expressing population (BrdU-SCGN/SCGN).

### Increased neuronal activity in SCGN-positive cells was observed in both models of olfactory impairments but was higher in PCD mice

Finally, we decided to analyze the nuclear activity of the SCGN-positive neurons, as an approximation of the functionality of these cells. For this purpose, the c-Fos marker was used, since monitoring its expression levels has been established as a reliable technique to identify neural populations of metabolically activated brain regions^26,27^.

**Figure 6:**
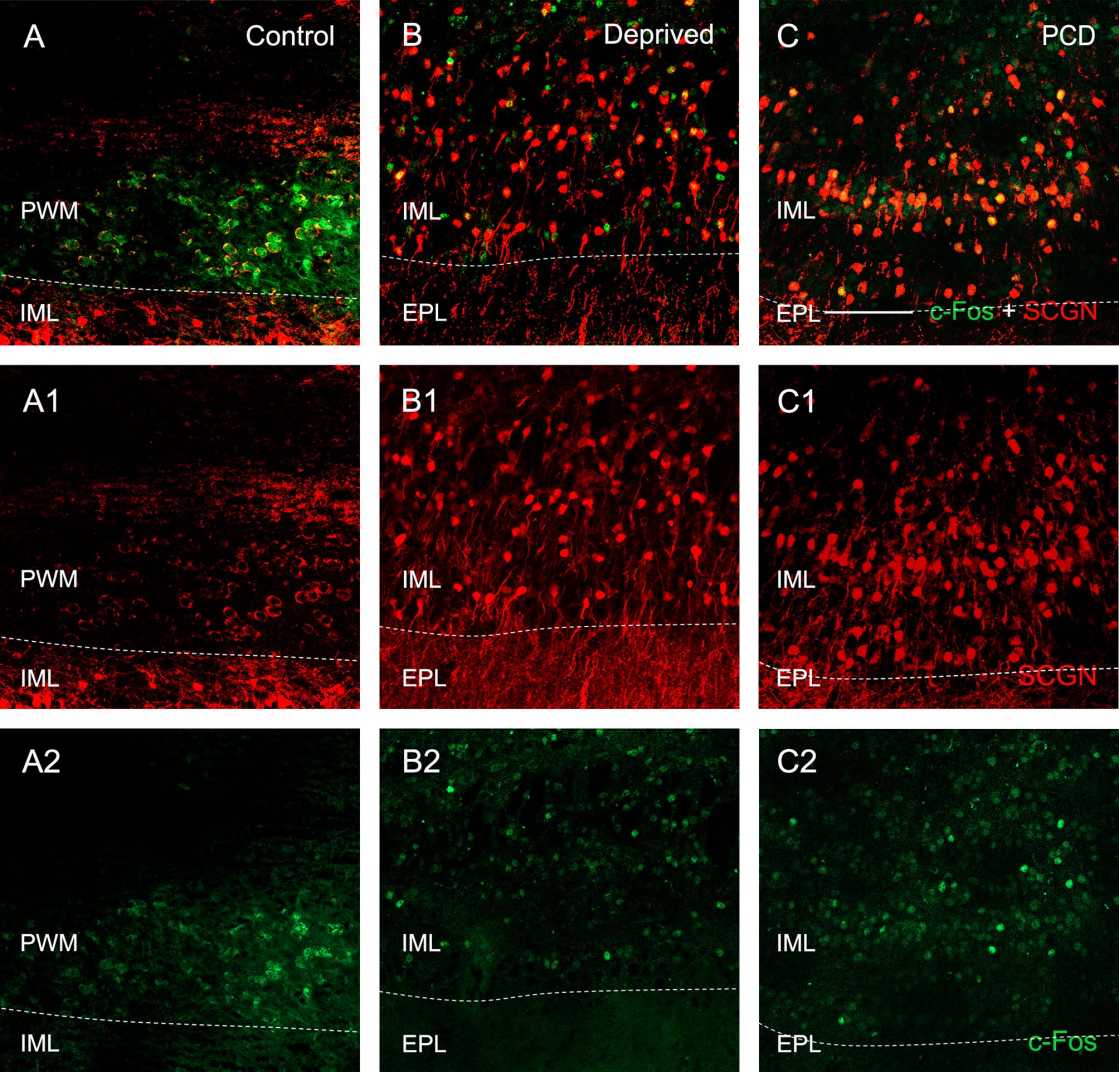
Expression of SCGN (red) and c-Fos (green) in control (**A**), deprived (**B**) and PCD (**C**) mice. The separate channels are also shown for SCGN (**A1**–**C1**) and c-Fos (**A2**–**C2**). In the control mice, c-Fos-positive cells are only observed in the periependymal white matter (PWM) and show a large nucleus and a small cytoplasm (**A**). In deprived (**B**) and PCD (**C**) mice, double immunopositive cells for c-Fos and SCGN appear in all strata, mainly in inframitral layers (IML) and the glomerular layer (not shown), being very scarce in the external plexiform layer (EPL). Scale bar 100 µm.

In the control mice, c-Fos-positive cells were only found in the periependymal white matter (n = 6; Fig. 6A). More specifically, c-Fos expression occasionally appeared in clusters of cells in the most caudal level, and only in some of the sections analyzed. Regarding their morphology, these c-Fos-positive cells of the periependymal white matter, when also co-expressed SCGN, presented large nuclei and scarce cytoplasm (Fig. 6A). By contrast, the other experimental groups showed c-Fos expression in all strata, especially in the GL and inframitral layers, but not in periependymal white matter (n = 6 each; Fig. 6B,C). Moreover, the morphology of double labeled c-Fos-SCGN cells in both deprived and PCD mice was different that the corresponding to control mice. Indeed, in these two experimental groups we observed a typical morphology of OB cells, being smaller, round and with a smaller nucleus compared to those found in control mice (Fig. 6).

Considering this inverse pattern of expression, and since it was greatly restricted in the control animals, we considered that both experimental paradigms radically changed the standard c-Fos labeling of an untouched OB. Then, we decided to quantify the extension of such change for each experimental condition. Since control animals presented a qualitatively different c-Fos expression, only the ratio of c-Fos expression in the SCGN-positive cells of deprived and PCD mice could be compared.

**Figure 7:**
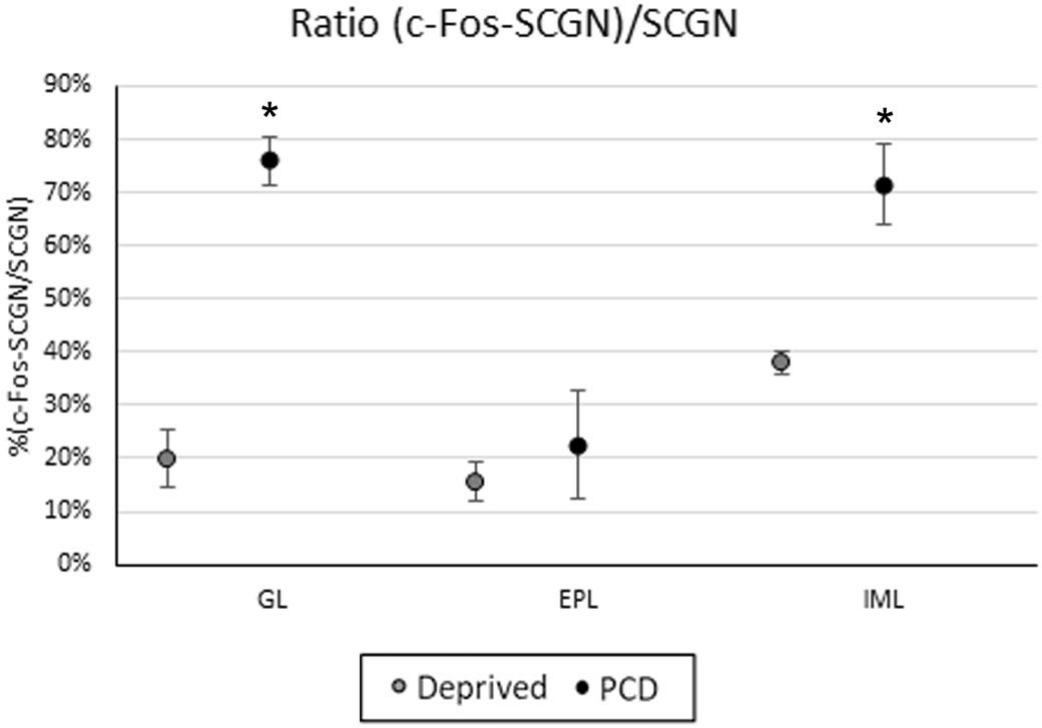
Quantification of c-Fos and SCGN colocalization in the OB of deprived (gray) and PCD (black) mice, expressed as doubly labeled cells in relation to the total SCGN-positive elements. As can be seen, there is a statistically significant increase of BrdU-SCGN co-expression in the glomerular layer (GL) and inframitral layers (IML) of PCD with respect to the deprived mice *p < 0.05, **p < 0.01.

In deprived mice, 24.52 ± 2.12% of SCGN-immunopositive cells colocalized c-Fos in the OB (data not shown). Within the separate strata, we observed 19.84 ± 5.43% of colocalization in the GL, 15.62 ± 3.56% in EPL and 38.10 ± 2.13% in the inframitral layers (Fig. 7). Furthermore, in PCD mice, c-Fos expression in SCGN-positive cells was higher, comprising a 56.70 ± 2.44% of the total SCGN-positive elements in the entire OB (data not shown). It was found that colocalization was 76 ± 4.52% in the GL, 23 ± 10% in the EPL, and 71.53 ± 7.72% in the inframitral layers (Fig. 7). A statistically significant increase was observed in c-Fos-positive cells in the OB of the PCD animals compared to the deprived mice in both GL and inframitral layers (*p* = 0.029 for both strata; Fig. 7).

## Discussion

### SCGN is related to olfactory inputs

Here, we examined how the density of SCGN-positive cells changed in the OB of two animal models with a partially impaired olfactory pathway. Interestingly, in deprived mice, despite the reduced number of TH-positive juxtaglomerular cells, the number of SCGN-positive cells increased, especially in the inframitral layers. In contrast, SCGN expression seemed to be relatively stable when severe bulbar circuitry alterations occurred, like the loss of the main projection neurons of the OB, the mitral cells, in PCD mice. This indicates that SCGN is related to olfactory inputs rather than to other olfactory functions.

Studies using deprived animals revealed that calcium-binding proteins exhibit different expression patterns and differential susceptibility to olfactory restriction. In particular, in these models, calbindin D-28K is reduced by 30% in the GL and parvalbumin decreases by 64% in the EPL, whereas calretinin expression is less affected^22^. Our results suggest that SCGN acts differently from other calcium-binding proteins, especially compared to calbindin D-28K and parvalbumin, since there is an increase in SCGN-positive cells after deprivation, and it mainly occurs in the GCL. Therefore, although all calcium-binding proteins seem to be regulated by the afferent activity associated with sensory experiences, the loss of activity caused by closing a nostril affects them in different ways.

Conversely, the expression of calcium-binding proteins such as calretinin, parvalbumin or calbindin in PCD mice do not suffers big modifications, especially in the GCL, almost intact^24,25^. These findings agree with those corresponding with SCGN, which does not present variations between PCD and control mice. Moreover, previous studies have shown that at P110 the PCD mutant mouse does not undergo noticeable changes in the GCL^13^, so it is logical to find this invariance in the expression of the calcium-binding proteins of this layer. Moreover, this relative stability of the GCL in PCD animals^13^ validates the density analyses performed in this layer at P110.

It is known that SCGN can act as a marker of neuronal damage^28^. However, as no variation in the density of SCGN-positive cells was observed in PCD mice (contrary to deprived animals), this finding supports the notion that changes in the expression of this protein are very specific and related to the type of damage, specifically the lack of orthonasal olfactory inputs.

Considering the differences between the rostral and caudal levels, there are several studies supporting the idea that the OB is not uniform. Indeed, the primary progenitors coming from the subventricular zone in adult animals are heterogeneous and predetermined to generate specific types of neurons^29^. Taking it into account, the different regions of OB have specialized interneuron populations and functions that can be considered different^30^. Various subtypes of interneurons form different connectivity maps and modes of experience-dependent plasticity in the OB, which may reflect their unique functional roles in information processing^31^. Besides, there are four additional subtypes of interneurons that differ in a very specific area of the OB, suggesting special functional contributions to OB circuits^32^. Taking into account the population of granule cells, there are several subtypes of cells that play different roles depending on their origin^3^. There are also differences in the GL, concerning atypical glomeruli that are neurochemically different to typical glomeruli. Thus, their interneurons can be considered different from those present in the typical glomeruli^33^. This variability of expression can also be observed in other molecules, such as G proteins, very important in the transduction pathway of sensory stimuli. The expression of these molecules varies throughout the rostro-caudal extension of the OB, as well as comparing the main and the accessory olfactory bulbs^34^. This asymmetric distribution correlates with the differential SCGN expression between caudal and rostral levels of the OB. Once again, our data support the hypothesis of a different functionality of calcium-binding proteins, not only considering the type of molecule^22^ or neuronal damage^28^, but also because of its rostral-caudal location.

Taking into account these data, we propose two possible opposing functions for this protein considering that the increase of SCGN-positive cells after deprivation lies in the GCL, and granule cells are responsible of the modulation of mitral cells activity^35–38^. On one hand, we propose that SCGN can reduce the activity of granule cells. In this case, the increase of SCGN in deprived animals would reduce the feedback inhibition produced by these interneurons into the mitral cells^36,37^. Therefore, the nervous impulses coming out from the OB would be maximized in a context of reduced sensory input stimuli, as a compensatory mechanism. On the other hand, we suggest an opposite function, considering that the SCGN increases the activity of granule cells. In this case, the compensation would produce an increase in the mitral cells inhibition by feedback^36,37^, achieving greater olfactory discrimination. Even considering both hypotheses, we can assume that SCGN is involved in the processing of olfactory information by modulating the interneurons action (this will be discussed later on together with the activity analysis of c-Fos). In any case, both hypotheses support strong compensatory characteristics of the OB functionality, as it happens when other alterations occur in this structure, not only in rodents^39^ but also in humans^40^.

### The increase in the density of SCGN-positive cells in deprived mice is not due to the arrival of newly formed cells

The neuroblasts that differentiate into interneurons have different patterns of expression that depend on its genotype^22^. In this sense, the pattern of SCGN expression is still unknown, and for this reason we analyzed the arrival of new cells to the OB (what is seen 15 days after BrdU injection) and the cell radial migration and differentiation (what is seen 30 days after BrdU injection).

The analysis of BrdU-SCGN colocalization showed no significant differences between the groups analyzed both 15 and 30 days after BrdU injection. Specifically, we did not found differences in the proportion of newly formed cells that differentiate into SCGN-positive cells (BrdU-SCGN/BrdU), nor in the proportion of the total population of SCGN-positive cells that were recently formed (BrdU-SCGN/SCGN) between control and deprived animals. Each calcium-binding protein shows different patterns of expression and localization in the OB. However, the neuroblast differentiation to SCGN was quite low in all cases studied.

Moreover, an accumulation of newly formed cells was described in the rostral migratory stream of deprived animals, which leads to a reduction in the number of cells that arrive to the OB and differentiate into interneurons^19^. Nonetheless, we observed not only the same ratio of newly formed cells in control and deprived mice, but also an increase of SCGN-positive cells in deprived animals. Therefore, all these data suggest that the increase of SCGN expression in deprived mice is due to protein overexpression in the interneurons of the OB, and not as a result of the arrival of new cells.

### The cellular damage produced by the different impairments entails an increase in c-Fos-positive cells

The nuclear activity was estimated by c-Fos expression. In control mice this expression was found in the periependymal white matter as occasional patches of cells with a large nucleus and a reduced cytoplasm labeled with SCGN. Their morphology and location suggest they could be new cells formed in the subventricular zone that migrate through the periependymal white matter into the OB^41^ and start to express SCGN^8^. If this hypothesis is assumed, the scarcity of these patches of putative SCGN-positive neuroblasts would agree with the results of the BrdU analysis. Hence, this pattern of c-Fos expression is greatly restricted in control animals, also being completely different from what we observed in deprived and PCD mice: much more c-Fos-positive cells with a wider extension. Accordingly, Matsuda et al. (1996) found a very low number of c-Fos expression in the OB of control mice whereas in animals subjected to chronic stress, this expression increased^42^. Altogether these data suggest that olfactory impairments radically change the basal expression of c-Fos of an untouched OB.

Regarding the experimental groups with olfactory impairments, our results showed an increased nuclear activity (in terms of c-Fos expression) in the SCGN-positive cells in all OB layers, being higher in PCD than in deprived mice. This difference in the ratio of c-Fos-expressing cells among SCGN-positive neurons could be partially explained by the dissimilarity of results concerning the inframitral layers of deprived and PCD animals: since the number of SCGN-positive cells are higher in deprived mice, the final ratio (c-Fos-SCGN/SCGN) could be lower. However, this reasoning cannot be applied to the GL, where SCGN expression is similar in both PCD and deprived animals. These data imply a general increase in c-Fos expression in PCD mice independently of variations in SCGN labeling, at least in part. Additionally, the severity of bulbar alteration should be pointed out. According to Briñón et al. (2001), olfactory deprivation produces a slight increase of c-Fos expression during the first days after deprivation, and this increase is more evident at longer deprivation times, when more c-Fos immunoreactive cells can be located in the GCL and the GL^10^. Considering that c-Fos is frequently used to detect pathogenesis in disorders of the central nervous system^43^, the differential expression of c-Fos under different impairments suggests a direct relationship between the level of expression and the severity of the disability. Then, since the c-Fos ratio of SCGN-positive cells is higher in PCD than in deprived mice, the damage that occurs in the former model (i. e., the loss of its main projection neurons) seems to be more severe than the impairments related to olfactory deprivation^13^, at least for the expression of the markers analyzed in this study.

Besides, in other animal models, like Tbr1 mice, an increase in c-Fos expression has beneficial effects against mutations that produce deficits in olfactory discrimination^31^. Hence, these changes in c-Fos expression may involve a strategy aimed at maintaining the highest level of ability to send or detect olfactory signals, since several studies associate the c-Fos expression with the cellular response to lesions, as well as to neuronal plasticity^44^. Taking this reasoning into account, at the beginning of this section two opposing hypotheses concerning the function of SCGN were discussed. Since a general increase of the nuclear activity of SCGN-positive cells was observed in deprived mice (in terms of c-Fos expression), it would support our second hypothesis, that is to say, SCGN would increase the activity of granule cells to enhance the mitral cells inhibition by feedback^36^, in an attempt to achieve greater olfactory discrimination.

## Conclusion

Our results suggest two different changes in the neurochemistry of the OB, probably acting as compensatory mechanisms. A reduction in the inputs (anterograde damage), as seen in deprived mice, provokes an increase in the density of SCGN-positive cells and a qualitative change (and increase) of c-Fos labeling. By contrast, output impairments (retrograde damage), like in PCD mice, only causes a c-Fos increase, but more noticeable than in the former experimental condition. Therefore, we could conclude that different compensatory mechanisms are put into motion depending on the type of alteration. The OB is extremely plastic, and the mechanisms that regulate this plasticity seem to be more complex than previously thought.

## Experimental procedures

### Animals

C57/DBA mice (Jackson ImmunoResearch Laboratories, UK) were housed at the animal facilities of the University of Salamanca at a constant temperature and humidity, with a 12/12 h photoperiod. They were fed with water and special rodent chow ad libitum (Rodent toxicology diet, B&K Universal G.J., S.L. Molins de Rei, Barcelona, Spain). Three groups of male mice were analyzed at P110: (1) PCD; (2) olfactory-deprived; and (3) controls (wild type animals; n = 6 each). In addition, four other groups, 10 deprived male mice and 10 control male mice, were used to analyze 5′-bromo-2′deoxiuridine (BrdU) expression (described below). All animals were housed, manipulated and sacrificed in accordance with current European (2010/63/EU, Recommendation 2007/526/CE) and Spanish Legislation (Law 32/2007, RD 53/2013); the experiments were approved by the Bioethics Committee of the University of Salamanca.

#### Naris occlusion

A unilateral olfactory occlusion was performed at P70, when the degeneration of the mitral cells in PCD mouse becomes evident^23^, using electrocoagulation and suture techniques on the group of deprived animals previously anesthetized with isoflurane (Zoetis Spain, Madrid, Spain). Once anesthetized, electrocoagulation was carried out by introducing an electrocautery into the right naris and applying 2 or 3 electrical pulses. Finally, topical antibiotics were applied to the wound to prevent infection. The integrity of the occlusion was checked every two days, which supported an optimal olfactory deprivation, confirmed afterwards by the immunolabeling of TH (see “Results” and later).

### Bromodeoxyuridine administration

To label proliferative cells, thymidine analog BrdU (Sigma Chemical Co., St. Louis, USA) was administered intraperitoneally, mixed with the thymidine synthesis inhibitor 5′-fluoro-2′-deoxyuridine (Sigma; 3 µg/g b.w.) in 0.1 M phosphate buffered-saline (PBS), pH 7.3. In order to analyze both migration and differentiation, control and deprived mice were injected with BrdU either 15 or 30 days before being sacrificed (n = 5 each). Three consecutive doses of BrdU were injected every 3 h into the animals of both groups, according to a previously described method^45^.

### Sacrifice and tissue processing

Mice were deeply anaesthetized with 10 µl/g of body weight of chloral hydrate (Prolabo, Fontenay-sous-Bois, France). Then, they were sacrificed at P110, when the degeneration of mitral cells in the PCD model is complete^23^, by intracardiac perfusion with 0.9% NaCl, followed by Somogyi’s fixative (4% w/v depolymerized paraformaldehyde and 15% v/v saturated picric acid in PBS). After perfusion, the animals’ brains were dissected out, divided into three blocks along the coronal plane using a mouse brain matrix and postfixed with the same fixative for 2 h.

The brain blocks were then rinsed for 2 h with 0.1 M phosphate buffer (PB), pH 7.4, and immersed in 30% (w/v) sucrose in PB until each block sank. After this cryoprotection treatment, 40 µm-thick coronal sections were obtained using a freezing-sliding microtome (Leica Frigomobil, Jung SM 2000, Nussloch, Germany). The sections were collected in six series in PB and stored at − 20 °C in an anti-freezing mixture of 30% glycerol (v/v) and 30% polyethylene glycol (v/v) in PB.

#### Immunolabeling

For immunolabeling, sections were rinsed in PBS (3 × 10 min) and incubated with 0.2% (v/v) Triton X-100 (Probus S.A., Barcelona, Spain), 5% (v/v) normal donkey serum (Vector laboratories, Burlingame, USA) and primary antibodies in PBS for 72 h at 4 °C. The primary antibodies, used either alone or in combination, were the following: rabbit anti-secretagogin (1:50,000; kindly gifted by Dr. L. Wagner), rat anti-BrdU (1:5000; Cat. No. ab6326, Abcam, Cambridge, UK), sheep anti-TH (1:5000; Cat. No. AB1542 Millipore Corporation, Burlington, USA) and mouse anti-c-Fos (1:1000; Cat. No. sc-271243 Santa Cruz Biotechnology, Dallas, USA). Finally, the sections were rinsed in PBS (3 × 10 min) and incubated for 1 h in darkness at room temperature with the corresponding secondary antisera: Cy3 conjugated donkey anti-rabbit IgG (Cat. No. 711–165-152), Cy2 conjugated donkey anti-rat IgG (Cat. No. 712–225-150) and/or Cy2-conjugated donkey anti-mouse IgG (Cat. No. 705–225-147; all of them at 1:500, Jackson). Ten minutes before the end of the incubation, 4′, 6′-diamidino-2-phenylindole (DAPI; 1:10,000 v/v; Sigma) was added to the medium to counterstain the cell nuclei. Finally, sections were rinsed in PBS (3 × 10 min) in the dark and mounted with coverslips using a freshly prepared anti-fade solution.

For BrdU detection, sections were rinsed in PBS (3 × 10 min) and then incubated for 1 h at 37 °C with 2 N HCl to allow DNA denaturation. After HCl incubation, tissues were rinsed in 0.1 M borate buffer (pH 8.5, 3 × 10 min) and then in PBS (3 × 10 min). Finally, the samples were incubated with a primary and secondary antiserum (as described above).

TH immunodetection was performed in order to determine the success of the deprivation. For this staining, an alternative system of visualization was employed. Sections were rinsed and incubated with primary antiserum for 72 h at 4 °C (as described above). Then, sections were rinsed in PBS (3 × 10 min) and incubated in 1:300 biotinylated sheep secondary antiserum (Cat. No. 713-065-003, Jackson) for 1 h at room temperature. Tissues were rinsed in PBS and incubated in avidin–biotin-peroxidase complex (Kit ABC; 1:200; Cat. No. PK-4000, Vector) for 60 min at room temperature. Finally, sections were rinsed three times in PBS, twice in 0.2 M Tris–HCl buffer (pH 7.6), and the reaction product was visualized by incubating the sections in 0.02% 3,3- diaminobenzidine (w/v) and 0.003% hydrogen peroxide (v/v) in 0.2 M Tris–HCl buffer (pH 7.6). Sections were mounted on a gelatinized slide and dehydrated with ethanol at increasing graduations and then rinsed with xylol and covered with Entellan (Millipore Corporation) and coverslips.

Specificity controls for these techniques were performed by removing either primary or secondary antibodies. No nonspecific staining was detected in any case.

### Quantitative analysis

All analyses were performed by the same person (L. P.-R.) by counting SCGN-positive cells and the other markers along three strata: GL, EPL and inframitral layers, which included the MCL, GCL and the periependymal white matter, based on previous studies^25^. Additionally, each stratum was studied separately in dorsal, ventral, medial and lateral sectors.

First, to determine the degree of deprivation, the number of TH-positive juxtaglomerular cells per glomerulus in control and deprived mice was estimated. For this purpose, all TH-immunolabeled round elements (cells) were count in two glomeruli of four sectors (dorsal, ventral, medial and lateral) of a section of the OB. Then, the mean number of cells/glomerulus of that eight glomeruli was calculated in each mouse, and finally, in each experimental group (n = 6 each).

For the analysis of SCGN expression, the study was carried out in two different rostro-caudal levels amongst the different experimental groups as previously described^46^. The section chosen for the analysis of the caudal level was the first one abutting to the accessory olfactory bulb, without containing it (Bregma 4.145 mm; Allen Brain Atlas^47^); for the rostral level was the most-rostral section in which all the OB layers were clearly defined (Bregma 5.345; Allen Brain Atlas^47^). Cell density was calculated by referring the number of SCGN-positive cells present to a random area of the four sectors described above (dorsal, ventral, medial and lateral), also containing all the strata (GL, EPL and inframitral layers). The cell count was performed using a microscope equipped with the Neurolucida (V8.23, MicroBrightField, Colchester, VT, USA) and Neuroexplorer systems (V4.70.3, MicroBrightField). For this counting, only complete (with visible nuclear) round cells were considered independently of the intensity of their staining (fibers and small cell fragments were discarded). Around 20 cells in the GL, 5 in the CPE and 100 cells in the inframitral layers of each analyzed sector were gathered. Then, the cell density (in terms of number of cells / mm^2^) was calculated for each strata of the four sectors. We also estimated the density of SCGN-positive cells in the different strata of the whole bulbar section (i.e. without considering sectors); for this purpose, the four values of cell number and surface were considered together. Once all these values of cell density were calculated, the mean for each experimental group (n = 6) was obtained.

Cell migration and differentiation were analyzed by administering BrdU either 15 days (for analyzing the arrival of new cells to the OB) or 30 days before sacrifice (for analyzing cell differentiation^25^); that is to say, 25 and 10 days after olfactory deprivation respectively. Quantification was performed in two complementary ways: on one hand, cells doubly labeled for both SCGN and BrdU were referred to the total BrdU-positive elements (ratio BrdU-SCGN/BrdU) to determine the proportion of newly formed cells that differentiate and express SCGN; and on the other, the same double-labeled cells were referred to the total SCGN-positive cells (ratio BrdU-SCGN/SCGN) to estimate the proportion of the total population of SCGN-positive cells that are newly formed. Both analyses were carried out in each stratum separately by counting around 30 elements in the GL, 5 in the CPE and 60 in the inframitral layers, with the same morphology (whole and round nuclei), independently of the level of their staining, in each of the four bulbar sectors (dorsal, medial, ventral and lateral). As describe above, the values of the whole OB were estimated considering together the cell numbers of the four sectors. Then, the mean of each group (control and deprived mice, n = 5) was calculated.

The activity of SCGN-positive cells was also estimated by counting the number of double-labeled cells for both c-Fos and SCGN cells and relating it to the total of SCGN-positive elements (ratio c-Fos-SCGN/SCGN). We counted in each bulbar sector around 20 cells in the GL, 5 cells in the CPE and 60 cells in the inframitral layers, with the typical nuclear morphology for c-Fos (round nuclei). The values for each stratum of the whole OB were calculated as described above, and then the mean of each group (PCD and deprived mice, n = 6) was obtained.

### Statistical analysis

For the statistical treatment of data, the non-parametric Kruskal–Wallis analysis with its post-hoc tests (when required) was performed for comparing the three groups, control, deprived and PCD, and to determine the possible differences in cell densities or ratios in both rostral and caudal levels. Since the OB may present regional differences^29^, these analyses were performed for each of the three strata (GL, EPL and inframitral layers), both in a general way (considering each OB section as a whole) as a first approximation, and in each sector (dorsal, ventral, lateral or medial) separately, for a more accurate regional analysis. Additionally, when only two experimental groups were compared (see results), the non-parametric Mann–Whitney U test was employed in the same conditions described above. Statistically significance was set at *p* < 0.05.

## Supplementary Information

Supplementary Table 1.

Supplementary Table Legends.

## Data Availability

The datasets generated during and/or analyzed during the current study are available from the corresponding author on reasonable request.

## Abbreviations

BrdU: 5′-Bromo-2′-deoxiuridine
DAPI: 4′-6-Diamidino-2-phenylindole
EPL: External plexiform layer
GCL: Granule cell layer
GL: Glomerular layer
MCL: Mitral cells layer
OB: Olfactory bulb
PB: Phosphate buffer
PBS: Phosphate-buffered saline
PCD: Purkinje cell degeneration
SCGN: Secretagogin
TH: Tyrosine hydroxylase
