# Supplementary material for: Secretagogin expression in the mouse olfactory bulb under sensory impairments
Source: Sci Rep. 2020 Dec 9;10:21533. doi: 10.1038/s41598-020-78499-5 (PMC7726155; doi:10.1038/s41598-020-78499-5)
Supplement: Supplementary file 1 — Supplementary Table 1. [file 41598_2020_78499_MOESM1_ESM.pdf]

Secretagogin expression in the mouse olfactory bulb under sensory impairments

Authors' Names and Institutions:

L. Pérez-Revuelta [1,2], P. G. Téllez de Meneses [1,2], M. López, J.G. Briñón[1,2], E. Weruaga[1,2], D. Díaz[1,2,CA] and J.R. Alonso[1,2]

1 Laboratory of Neuronal Plasticity and Neurorepair, Institute for Neuroscience of Castile and Leon (INCyL), University of Salamanca, Salamanca, 37007, Spain.

2 Institute of Biomedical Research of Salamanca, IBSAL, Salamanca, 37007, Spain.

[CA] **Corresponding author**:

Dr. D. Díaz, Institute for Neuroscience of Castile and Leon, Universidad de Salamanca, C/ Pintor Fernando Gallego, 1, E-37007, Salamanca, Spain.

E-mail: ddiaz@usal.es; phone: +34 923 294500 ext. 5326

CAUDAL

		Control		Deprived			PCD		
		Mean	SEM	Mean	SEM	*p*-value	Mean	SEM	*p*-value
**GL**	Whole OB	1047.35	128.10	1519.31	175.41	0.165	1231.15	116.74	0.201
	Dorsal	982.13	153.98	1581.32	194.51	0.066	946.32	158.24	1.000
	Medial	886.38	160.45	1635.33	172.90	0.183	1318.99	203.89	0.136
	Ventral	934.69	271.25	1506.97	138.03	0.138	1316.27	210.64	0.149
	Lateral	1553.09	214.32	1510.88	185.18	0.740	1348.77	129.92	0.522
**EPL**	Whole OB	35.45	8.12	59.27	8.82	0.118	112.39	31.36	0.068
	Dorsal	5.95	5.95	76.01	28.98	0.239	105.57	48.78	0.180
	Medial	31.15	16.41	56.86	20.38	0.221	120.52	43.48	0.131
	Ventral	16.01	9.83	37.54	29.43	0.102	131.30	52.64	0.062
	Lateral	88.40	17.20	68.58	21.21	0.745	88.75	32.74	0.522
**IML**	Whole OB	929.50	94.94	1749.30	66.06	**0.005**	1004.52	114.76	0.831
	Dorsal	1044.14	155.99	1750.29	113.23	**0.009**	1046.93	145.56	0.624
	Medial	827.62	96.62	1714.21	87.66	**0.007**	1130.06	127.85	0.136
	Ventral	871.92	87.07	2017.40	191.82	**0.008**	955.27	115.70	0.394
	Lateral	914.34	171.98	1570.67	74.81	**0.025**	955.04	166.40	0.831

ROSTRAL

		Control		Deprived			PCD		
		Mean	SEM	Mean	SEM	*p*-value	Mean	SEM	*p*-value
**GL**	Whole OB	1386.43	251.08	2327.03	316.91	0.053	2447.31	251.34	0.053
	Dorsal	1622.30	634.76	2097.23	330.84	0.053	2195.82	490.87	0.180
	Medial	2150.25	708.25	2451.41	591.99	0.881	2274.25	355.34	0.297
	Ventral	1068.40	439.33	2112.04	713.01	0.297	1919.60	450.00	0.101
	Lateral	822.01	491.61	2647.43	505.26	0.071	3342.50	878.03	0.071
**EPL**	Whole OB	150.13	21.82	159.69	60.88	0.655	237.70	92.55	0.881
	Dorsal	262.89	131.56	292.76	216.97	0.647	300.07	163.99	0.764
	Medial	93.75	76.55	167.05	41.72	0.131	97.50	66.15	0.864
	Ventral	43.13	43.13	109.58	52.53	0.285	315.03	95.87	0.266
	Lateral	168.36	138.73	69.39	16.90	0.439	256.10	74.17	1.000
**IML**	Whole OB	1506.10	232.71	2045.40	135.55	0.101	1812.31	129.33	0.180
	Dorsal	1352.39	262.65	2160.39	311.71	0.101	2162.91	317.43	0.101
	Medial	1504.22	251.99	1976.92	155.06	0.180	2152.09	159.13	0.053
	Ventral	1606.93	449.04	2225.49	314.00	0.180	1698.74	92.42	0.655
	Lateral	1745.42	454.67	1818.81	81.57	0.655	1235.48	265.17	0.456
