# Supplementary material for: Secretagogin expression in the mouse olfactory bulb under sensory impairments
Source: Sci Rep. 2020 Dec 9;10:21533. doi: 10.1038/s41598-020-78499-5 (PMC7726155; doi:10.1038/s41598-020-78499-5)
Supplement: Supplementary file 2 — Supplementary Table Legends. [file 41598_2020_78499_MOESM2_ESM.docx]

**Supplementary Table 1**. Raw data of the density of SCGN-positive cells (cells/mm^2^) in caudal and rostral levels of the control, deprived and PCD mice. The data are distributed in glomerular layer (GL), external plexiform layer (EPL) and inframitral layers (IML), as well as in the whole OB or in the four sectors analysed (dorsal, medial, ventral and lateral). The table also shows as the *p-value* obtained after comparing the experimental groups with controls; the values that are statistically significant (*p* > 0.05) are shown in bold.
